# A national study of moral distress among U.S. internal medicine physicians during the COVID-19 pandemic

**DOI:** 10.1371/journal.pone.0268375

**Published:** 2022-05-16

**Authors:** Jeffrey Sonis, Donald E. Pathman, Susan Read, Bradley N. Gaynes

**Affiliations:** 1 Department of Social Medicine, University of North Carolina at Chapel Hill, Chapel Hill, North Carolina, United States of America; 2 Department of Family Medicine, University of North Carolina at Chapel Hill, Chapel Hill, North Carolina, United States of America; 3 Cecil G. Sheps Center for Health Services Research, University of North Carolina at Chapel Hill, Chapel Hill, North Carolina, United States of America; 4 Research Center, American College of Physicians, Philadelphia, Pennsylvania, United States of America; 5 Department of Psychiatry, University of North Carolina at Chapel Hill, Chapel Hill, North Carolina, United States of America; 6 Department of Epidemiology, University of North Carolina at Chapel Hill, Chapel Hill, North Carolina, United States of America; PLoS ONE, UNITED STATES

## Abstract

**Background:**

There have been no studies to date of moral distress during the COVID-19 pandemic in national samples of U.S. health workers. The purpose of this study was to determine, in a national sample of internal medicine physicians (internists) in the U.S.: 1) the intensity of moral distress; 2) the predictors of moral distress; 3) the outcomes of moral distress.

**Methods:**

We conducted a national survey with an online panel of internists, representative of the membership of the American College of Physicians, the largest specialty organization of physicians in the United States, between September 21 and October 8, 2020. Moral distress was measured with the Moral Distress Thermometer, a one-item scale with a range of 0 (“none”) to 10 (“worst possible”). Outcomes were measured with short screening scales.

**Results:**

The response rate was 37.8% (N = 810). Moral distress intensity was low (mean score = 2.4, 95% CI, 2.2–2.6); however, 13.3% (95% CI, 12.1% - 14.5%) had a moral distress score greater than or equal to 6 (“distressing”). In multiple linear regression models, perceived risk of death if infected with COVID-19 was the strongest predictor of higher moral distress (β (standardized regression coefficient) = 0.26, p < .001), and higher perceived organizational support (respondent belief that their health organization valued them) was most strongly associated with lower moral distress (β = -0.22, p < .001). Controlling for other factors, high levels of moral distress, but not low levels, were strongly associated (adjusted odds ratios 3.0 to 11.5) with screening positive for anxiety, depression, posttraumatic stress disorder, burnout, and intention to leave patient care.

**Conclusions:**

The intensity of moral distress among U.S. internists was low overall. However, the 13% with high levels of moral distress had very high odds of adverse mental health outcomes. Organizational support may lower moral distress and thereby prevent adverse mental health outcomes.

## Introduction

Moral distress in health care is defined as the discomfort that health workers feel when they are prevented, by persons, institutions or situations, from doing what they believe is morally right [1–3]. It is different from moral uncertainty, when a health worker is uncertain about the ethically correct action to take in the context of a moral dilemma.

Moral distress was initially reported among nurses, but it has been described in physicians and other health workers as well [3, 4]. It is an important issue for health workers because it is associated with anxiety and guilt [5], depressive symptoms [6], and burnout and job attrition [3].

Early in the COVID-19 pandemic, multiple observers raised concerns about the potential for moral distress among health workers during the pandemic due to resource limitations, policies that promote safety but inhibit patient-centered care, and the need for health workers to weigh risks to self against the professional duty to provide the best care possible [7, 8].

There is now a growing body of empirical research on moral distress during the COVID-19 pandemic. Studies of moral distress related to the COVID-19 pandemic have reported that approximately 60% to 80% of health care workers have experienced at least some situations that generate moral distress [9–13]. However, the intensity (severity) of moral distress among health workers during the pandemic, in those studies that reported mean moral distress levels, has been found to be low in most [11, 13–21] though not all [22–24] studies. Among these studies, some assessed the intensity of moral distress for specific situations [15, 20], some assessed both the frequency and intensity of moral distress for specific situations [13, 14, 17, 18, 21, 22, 24, 25] and others assessed it with a global measure of moral distress without anchoring the assessment to specific potentially-morally-distressing situations [11, 19, 23]. The systematic reviews by Gianetta and colleagues [1] and by Tian and colleagues [26] provide comprehensive discussions of instruments that have been used to measure moral distress.

Multiple studies have attempted to identify individual and organizational factors that are associated with moral distress during the COVID-19 pandemic because those clues may lead to interventions to prevent it or mitigate its impact. Of the studies on moral distress conducted during the COVID-19 pandemic that used standard instruments to measure moral distress [11, 13–25], frequency of exposure to patients with COVID-19 was positively associated with moral distress [16, 19, 25], and adequacy of personal protective equipment [16, 17] and a positive ethical climate in the health organization [17] was negatively associated However, the effects of other individual characteristics, such as perceived risk of developing COVID-19 or risk of dying, if infected, on moral distress are unknown as are the effects of organizational characteristics, such organizational support of their health workers and leadership communication during the pandemic. In addition, it is unknown whether punitive organizational policies during the pandemic, such as sanctioning workers who speak out about COVID-19 safety, cause moral distress. Determination of these effects may help identify individuals at risk of high moral distress and organizational characteristics that can be modified to reduce moral distress.

Several studies during the COVID-19 pandemic have demonstrated that moral distress is associated with adverse mental health. Specifically, studies have demonstrated associations between moral distress and screening positive for anxiety [17, 19–23, 25], depression [17, 19–23, 25], burnout [17, 23] and PTSD [12, 17]. However, no studies have evaluated the impact of specific levels of moral distress (low, moderate, high) during the pandemic on adverse mental health outcomes. This is important because the information can be used to determine whether interventions to reduce moral distress should be aimed at all physicians with any degree of moral distress or only at physicians with moral distress levels that are associated with adverse outcomes.

Of the extant quantitative studies of moral distress during the COVID-19 pandemic, most used convenience sampling with indeterminate response rates [9, 10, 16–23, 25]. Two sampled defined populations but reported response rates less than 20% [11, 13]. Both factors raise concerns about the possibility of selection bias. Most of the studies were based on non-U.S. samples [9, 10, 13–15, 17–23, 25]; applicability to the United States, given differences in health systems, and organizational responses to the pandemic, is unclear. The studies based on U.S. samples [13, 16, 24] assessed moral distress among health workers at one [13, 16] or two [24] medical centers in the Northeast United States and the findings may not be applicable to physicians throughout the U.S.

We conducted a national study of Internal Medicine physicians (internists) in the U.S. to: 1) assess the frequency of compromised patient care due to resource limitations during the COVID-19 pandemic and the intensity of moral distress; 2) identify individual and organizational risk and preventive factors for moral distress intensity; and 3) assess the effect of moral distress on physicians’ mental health (generalized anxiety, depression, posttraumatic stress disorder (PTSD)), burnout and intention to leave direct patient care. More precisely, our third aim was to assess whether higher intensity of moral distress and whether specific levels of moral distress (low, moderate, high, each compared to none) were associated with adverse mental health.

## Materials and methods

### General

This study was part of larger study that also assessed the prevalence of adverse mental health outcomes among internists during the COVID-19 pandemic [27]. The study design was cross-sectional.

### Participants and survey administration

Details on participants and survey administration are reported elsewhere [27]. In brief, we conducted an online survey with internal medicine physicians who were members of the American College of Physicians (ACP) Insider Research Panel, an online study panel representative of the ACP membership. ACP is the largest medical specialty organization in the U.S. Panel members who provided direct clinical care at least 10% of their time were eligible to participate in this survey. The survey was open for 23 days, from September 15 to October 8, 2020, eight months into the pandemic, shortly after the start of the third surge in the United States. The study was deemed exempt by the University of North Carolina at Chapel Hill IRB (Study #: 20–0881). Informed consent was not required but a 700-word information sheet about the study’s goals and protections was provided to eligible respondents before they decided whether to participate.

### Key measures

#### Moral distress

Moral distress was measured with the Moral Distress Thermometer [28], a one-item visual-analog scale that defines moral distress and then asks respondents to rate the amount of moral distress they have experienced in the previous two weeks, rated from 0, “none” to 10, “worst possible [28]”. Please see Supporting Information for a representation of how the instrument appeared to survey respondents. The Moral Distress Thermometer has good convergent and discriminant validity and has been used to measure moral distress in nurse and physician samples [4]. Unlike some instruments measuring moral distress [29, 30], the Moral Distress Thermometer is not anchored to specific situations that cause moral distress [1]. Several studies have shown that compromised patient care related to resource limitations is one of the situations most likely to be associated with moral distress during the pandemic [11, 13]. Accordingly, following the assessment of moral distress, respondents were asked to rate how often they experienced “compromised patient care due to lack of resources/equipment/bed capacity” during the two weeks when the COVID-19 pandemic was at its worst in their health care organization. The five response options ranged from 1, “never” to 5, “very frequently”.

#### Potential predictors of moral distress

We assessed potential predictors of the intensity of moral distress from two broad categories: risk of exposure to COVID-19 and its consequences, and organizational factors related to the safety and support of health care workers.

Risk of exposure to COVID-19 was measured with the following variables: number of patients seen face-to-face with suspected or confirmed COVID-19 in the previous two weeks, inpatient clinical care (versus outpatient or both inpatient and outpatient), Internal Medicine subspecialty at high risk of exposure to patients with COVID-19 (Hospital Medicine, Infectious Disease, Pulmonary Medicine, Critical Care, Emergency Medicine versus all other subspecialties), perceived risk of developing COVID-19 and perceived risk of dying, if infected with COVID-19.

Organizational factors related to the safety and support of health care workers included perceived adequacy of personal protective equipment, perception of how well leaders in the respondent’s health organization listened to health worker concerns regarding COVID-19 (rated on a 5-point scale) and perceived level of organization support. Perceived organizational support was measured with a four-item scale, adapted from Eisenberger’s Perceived Organizational Support (POS) Scale, designed to measure the degree to which the respondent believed that their health organization “values their contributions and cares about their well-being” [31]. POS scores ranged from 4 to 20. We also included one dichotomously-scaled item on punitive leadership actions during the pandemic: “Do you know of any health care workers at your organization who have been warned or sanctioned for refusing assigned deployment or speaking up about worker / patient safety related to the COVID-19 pandemic?”

#### Consequences of moral distress

Mental health outcomes (generalized anxiety, depression, PTSD) and burnout were measured with short screening scales. Positive tests indicate probable disorders but not formal diagnoses. Generalized anxiety was measured with the GAD-2 [32]. A score of 3 or greater on the GAD-2, which ranges from 0 to 6, was considered a positive test [32]. Depression was measured with the PHQ-2 [33]. A positive test was defined as a score of 3 or greater (range 0–6) [33]. A positive screening test for PTSD was defined as a score of 6 or greater on the on a four-item scale based on the PCL-5 (range, 0–16) [34]. The three instruments to measure mental health outcomes were anchored to “the past two weeks,” matching the timing anchor for the moral distress thermometer. Burnout was measured with the single-item measure of emotional exhaustion, which performs similarly to the 22-item Maslach Burnout Inventory [35]. High burnout was defined, as in other studies, as feeling burned out from work once a week or more often, i.e., 4 or greater response value, range 0–6 [35].

Intention to leave clinical practice was measured using the single item, “What is the likelihood you will leave direct patient care in the next five years”, rated on a scale from 1very low to 5very high [36].

### Data analysis

#### Weighting of sample data

There were small but non-trivial differences between the age-gender and age-race/ethnicity composition of the sample and the ACP Insider Research Panel. We used raking [37] and post-stratification weighting [38] to make the age-gender and age-race/ethnicity distributions in the sample comparable to those in the ACP Insider Research Panel. All analyses were weighted and the sum of the weights equaled the sample total, i.e., N = 810.

#### Missing data

Missing data were uncommon. Four covariates had greater than 5% missing data: risk of death if infected with COVID-19 (14.2%), risk of COVID-19 infection (13.0%), ownership of the health organization (13.0%) and availability of personal protective equipment (5.1%). Missing data were assumed to be missing at random [39]. Descriptive statistics are based on non-missing responses for all variables. Full information maximum likelihood [39] in Mplus 8.5 [40] was used as the method of estimation for all regression modeling to address missing values in predictors.

#### Analyses of the research questions

To determine the intensity of moral distress and the frequency of compromised patient care due to lack of resources/equipment/bed capacity, we calculated weighted sample means and frequencies, with their 95% confidence intervals (CIs). The association between frequency of compromised patient care during the worst two weeks of the pandemic and the severity of moral distress during the two weeks prior to taking the survey was assessed with weighted Spearman correlation coefficient. We assessed the association between high frequency of compromised care due to resource limitations (rated 4, “commonly” or 5, “very frequently”) with high intensity of moral distress (greater than or equal to 6 on the 10-point scale) with the bivariate odds ratio and its 95% CI.

Linear regression was used to identify predictors of moral distress intensity [41]. We assessed the association between each predictor and moral distress, treated as a continuous dependent variable, controlling for potential confounding factors (shown in the first two columns in Table 1) and the association between each predictor and moral distress, controlling for potential confounding factors and all other predictors (shown in the last two columns in Table 1). To be able to compare the strength of the associations between predictors and moral distress, we reported associations as standardized regression coefficients [41, 42].

**Table 1:** Associations between predictors and moral distress.

	Multivariable, adjusted for demographic covariates^a^		Multivariable, adjusted for demographic covariates and all predictors^b^	
Predictor	β ^c^	*p-value* ^d^	β	*p- value*
Exposure to COVID-19				
Site of clinical care (inpatient vs. outpatient or both)	-0.05	0.15	-0.05	0.29
High-risk clinical subspecialty	0.01	0.90	-0.01	0.84
Number of patients with COVID seen face-to-face in previous two weeks	0.23	< .001	0.15	< .001
Perceived risk of developing COVID-19^e^	0.18	< .001	-0.01	0.97
Perceived risk of dying, if infected with COVID-19^e^	0.37	< .001	0.27	< .001
Organizational factors				
Adequacy of access to personal protective equipment	-0.26	< .001	-0.09	0.02
Leadership that listened to health workers regarding COVID-19^e^	-0.28	< .001	-0.03	0.54
Perceived organizational support scale	-0.35	< .001	-0.22	< .001
Hospital ownership (private vs. public)	-0.01	0.79	-0.01	0.97
Respondent knew of health workers at their organization who were warned or sanctioned for speaking up about COVID-19 safety	0.16	< .001	0.01	0.87

^a^Each model included the predictor and the following demographic covariates: age category, number of family members living at home, total number of clinical hours in the past week, gender, region of the United States of primary clinical practice (coded as three indicator variables), race/ethnicity (coded as four indicator variables).

^b^One model that included the demographic covariates and all of the predictors.

^c^β denotes standardized regression coefficient.

^d^p-values based on Z test: (parameter estimate / standard error)

To determine the association between moral distress and the dichotomized outcomes of interest (anxiety, depression, PTSD, burnout, intention to leave patient care), we used multivariable logistic regression, adjusting for potential confounding factors [43]. First, to assess whether there was a dose-response relationship between moral distress and the five adverse outcomes, we entered moral distress as an ordinal four-category independent variable in the logistic regressions. It was entered as an ordinal variable, rather than as a continuous variable, because it did not meet the linearity in the logit assumption of logistic regression as a continuous variable for all outcomes. [43]. Cutoff points for moral distress categories were based on the words used to anchor numerical response categories on the moral distress thermometer: none (0, “None”); low (1–2; “Mild”); moderate (3–5; “Uncomfortable); high (6–10; “Distressing”, “Intense”, or “Worst Possible”).

Second, to assess the association between specific categories of moral distress and the five adverse outcomes, we conducted separate logistic regressions comparing mild, moderate and high levels of moral distress to no moral distress for each of the five adverse outcomes.

Individual factors, such as demographic characteristics and risk factors for exposure to COVID-19, were considered potential confounders of the moral distress / outcome associations and were included in the models but organizational factors, such as perceived organizational support, were not considered potential confounders and were not included in the models. A two-sided p-value less than 0.05 was considered statistically significant.

## Results

### Sample characteristics

Of the 2,145 eligible panel members, 37.8% (N = 810) responded. A little less than half (45%) of the respondents were over the age of 45 and most (60%) were male. Almost one-third (29%) were in subspecialties at particularly high risk of exposure to COVID-19 [27]. Respondents reported a mean of 7.4 (95% CI, 6.2–8.6) patients with suspected or confirmed COVID-19 seen face-to-face in the previous two weeks. There were minimal differences between the ACP Panel and the weighted sample in the age-gender and age-ethnicity/race distributions [27].

### Moral distress intensity and prevalence of compromised patient care

**Fig 1:**
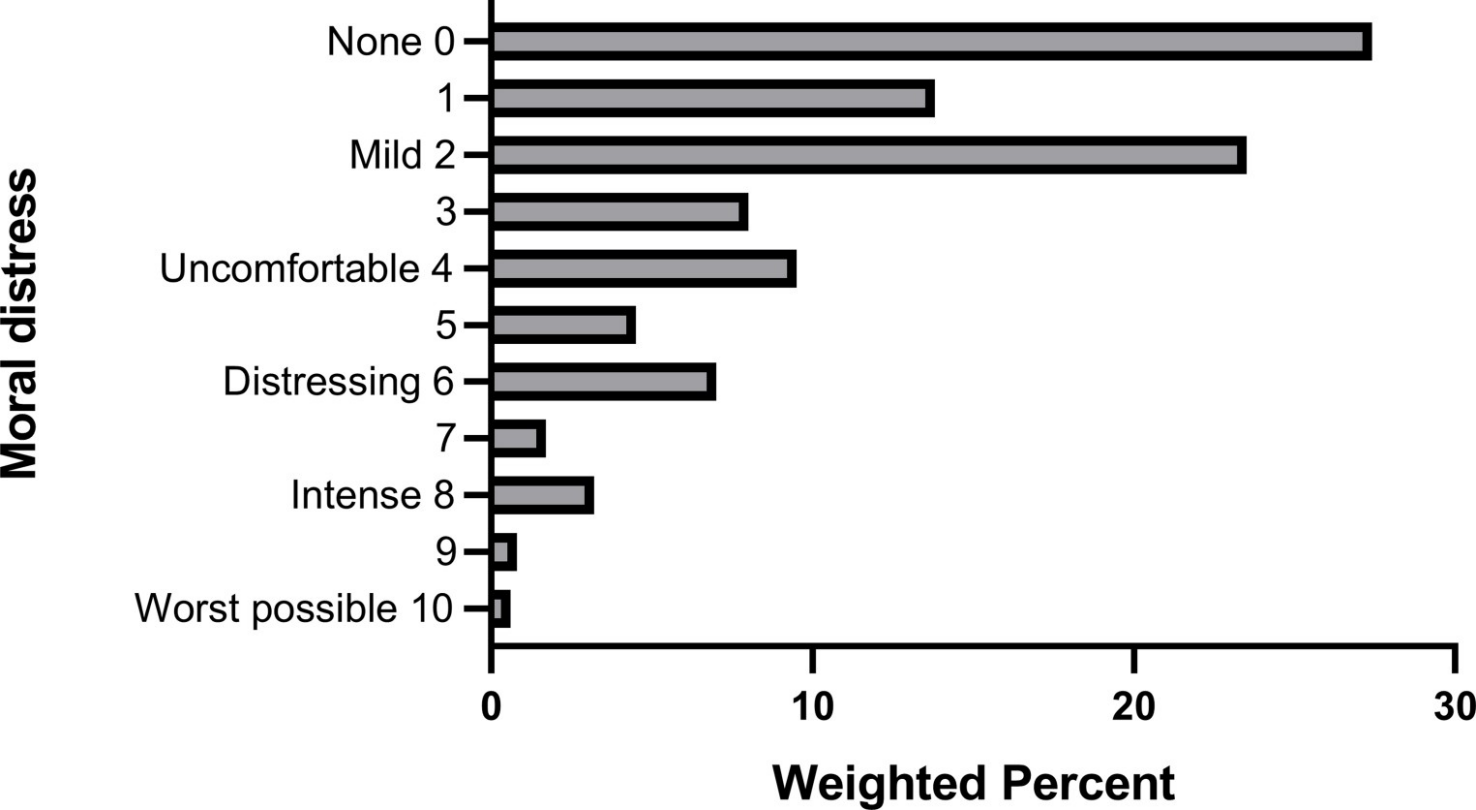
Intensity of moral distress in the past two weeks.

The mean moral distress score from work experiences in the two weeks prior to the survey was 2.4 (95% CI, 2.2–2.6). More than 4 out of 5 respondents (82.2%, 95% CI 81.0%-83.4%) had scores less than 4 (“uncomfortable”) but approximately 1 in 8 respondents (13.3%, 95% CI, 12.1% - 14.5%) had a moral distress score of 6 (“distressing”) or greater (Fig 1).

**Fig 2:**
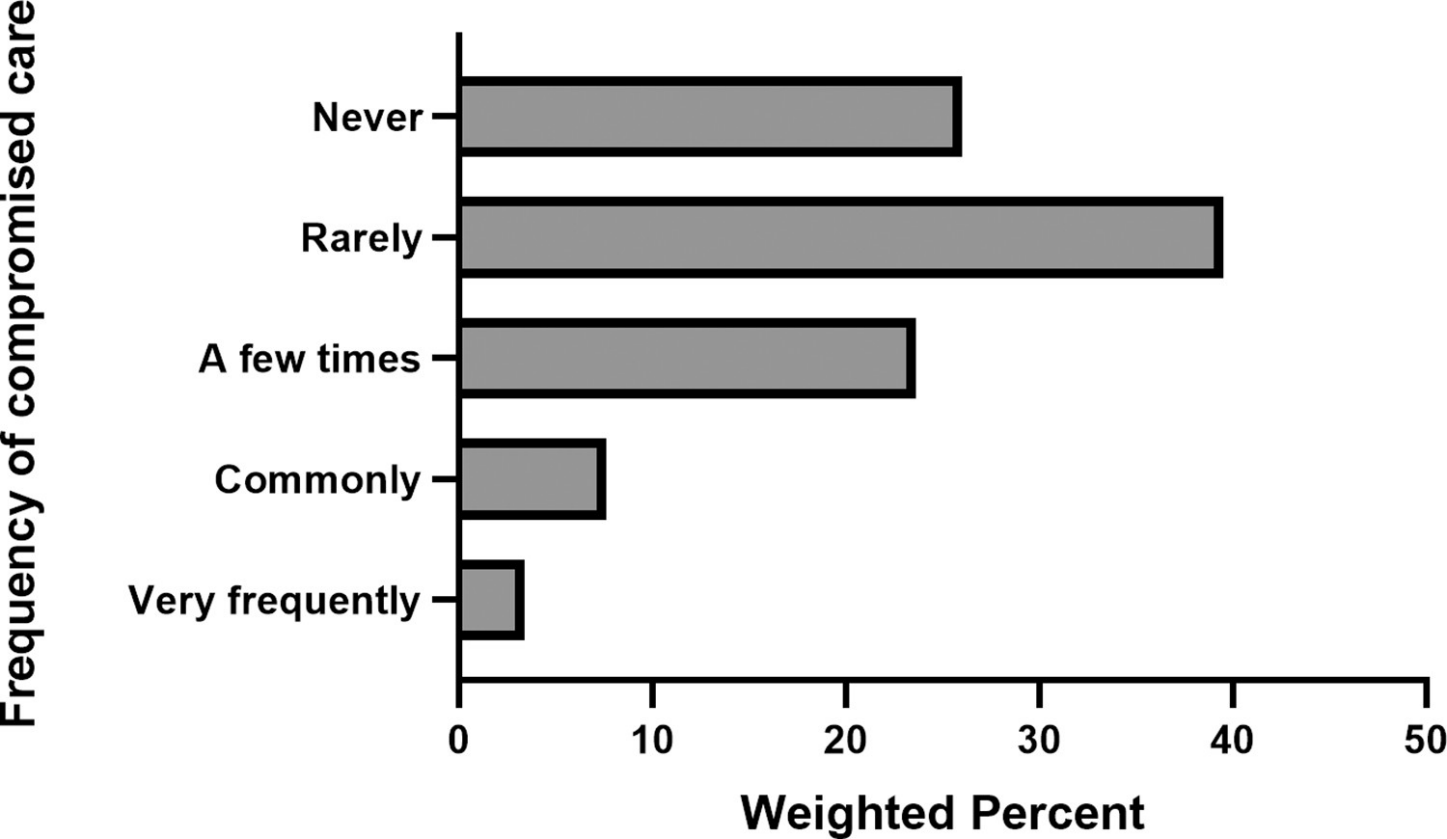
Frequency of compromised patient care due to resource limitations during the worst two weeks of the COVID-19 pandemic.

Nearly 3 in 4 respondents (74.0%, 95% CI, 71.1% - 76.9%) reported that they had experienced compromised patient care due to lack of resources at least once during the two weeks when the COVID-19 pandemic was at its worst in their health organization. Most respondents (89.0%, 95% CI, 86.9% - 91.1%), reported that they had experienced compromised care due to resource limitations a few times or less, including 26% who reported none (Fig 2). However, 11.0% (95% CI, 8.9% - 13.1%) experienced compromised patient care commonly or very frequently.

There was a moderate correlation between the frequency of experiencing compromised patient care during the worst two weeks of the pandemic locally and moral distress in the two weeks prior to the survey, r_s_ = 0.32, 95% CI, 0.26–0.38. Compared to respondents who reported experiencing compromised patient care due to resource limitations a few times or less during the two worst weeks of the pandemic, those who reported experiencing it commonly or frequently had much higher odds (OR 3.6, 95% CI, 2.1–6.1) of high levels of moral distress in the past two weeks.

### Predictors of moral distress

Perceived risk of developing COVID-19 and reported knowledge of health care workers at the respondent’s organization who were warned or sanctioned for speaking out on COVID-19 safety were associated with higher moral distress in models adjusting for confounding factors but were not independently predictive of moral distress in the multivariable model that included confounding factors and all of the other predictors (Table 1). Having leaders who listened to health workers regarding COVID-19 was associated with lower moral distress in models adjusting for confounding factors but was not independently predictive of moral distress in the multivariable model that included all of the other predictors (Table 1).

In a multivariable linear regression model, adjusting for covariates and all other predictors, two COVID-19 risks—the number of patients seen face-to-face with COVID-19 in the previous two weeks (β (standardized regression coefficient) = 0.15, 95% CI, 0.08–0.23) and perceived risk of dying if infected with COVID-19 (β = 0.27, 95% CI, 0.18–0.35)—were independently and positively associated with moral distress (Table 1). Two organizational factors—perceived adequacy of access to personal protective equipment (β = -0.09, 95% CI, -0.01 - -0.17) and perceived organizational support (β = -0.22, 95% CI, -0.12 - -0.32)—were independently and negatively associated with moral distress (Table 1). Based on the magnitude of the standardized regression coefficients, perceived risk of dying if infected with COVID-19 was the strongest predictor associated with higher moral distress and perceived organizational support the strongest predictor associated with lower moral distress.

### Association between moral distress and mental health, burnout and intention to leave patient care

There was a strong dose-response relationship between moral distress, coded ordinally, and each of the outcomes: for anxiety (adjusted odds ratio (aOR), 2.4, 95% CI, 1.9–3.1); for depression, aOR 2.0 (95% CI, 1.6–2.6); for PTSD, aOR 2.8 (95% CI, 2.2–3.8); for burnout, aOR 2.0 (95% CI, 1.7–2.4); for intention to leave patient care, aOR 1.6 (95% CI, 1.3–1.9).

However, as shown in Table 2, there were null associations between low levels of moral distress, compared to none, and all outcomes except anxiety, for which there was a weak association (aOR 2.4, 95% CI, 1.1–5.6). There were moderate to strong associations (aOR 2.1 to 6.6) between moderate levels of moral distress and the outcomes, except intention to leave patient care, which was not associated (aOR 1.4, 95% CI, 0.7–2.4). There were strong to very strong associations (aOR 3.0 to 11.5) between high levels of moral distress and all of the outcomes.

**Table 2:** Logistic regression associations between moral distress and mental health, burnout and intention to leave patient care.

	Anxiety	Depression	PTSD	Burnout	Intention to leave patient care
	aOR^a^^,^^b^ (95% CI)	aOR (95% CI)	aOR (95% CI)	aOR (95% CI)	aOR (95% CI)
Moral Distress					
None	Reference	Reference	Reference	Reference	Reference
Low	2.4 (1.0–5.5)	1.1 (0.5–2.4)	1.8 (0.6–5.0)	1.2 (0.7–1.9)	1.1 (0.7–1.9)
Moderate	4.9 (2.0–11.6)	2.4 (1.0–5.3)	5.6 (2.1–14.6)	2.1 (1.2–3.5)	1.1 (0.6–2.0)
High	10.4 (4.4–24.7)	4.3 (1.9–9.8)	11.5 (4.2–31.5)	7.3 (4.0–13.6)	3.0 (1.5–5.7)

^a^Odds ratios adjusted for age category, number of family members living at home, number of patients seen face-to-face in past week (coded as four-category variable), perceived risk of being infected with COVID-19, perceived risk of dying, if infected with COVID-19, gender, region of the United States (coded as three indicator variables), race/ethnicity (coded as four indicator variables).

^b^All models were run using full information maximum likelihood in Mplus 8.5.

## Discussion

To our knowledge, this is the first national study of moral distress related to the COVID-19 pandemic and its predictors and outcomes among physicians in the United States. There were five main findings. First, the average intensity of moral distress among internists was relatively low. However, 13.3% had moral distress scores that were in the distressing, intense or worst possible range. Second, nearly three in four respondents experienced at least one episode of compromised care due to resource limitations during the two worst weeks of the pandemic in their local communities. Most respondents (89%) experienced compromised care due to resource limitations a few times or less but 11% experienced it commonly or very frequently. Third, frequency of compromised care during the worst two weeks of the pandemic in the respondents’ local communities was moderately associated with current moral distress intensity. Fourth, in multivariable models, two factors related to exposure to COVID-19 (number of patients seen face-to-face with COVID-19 and perceived risk of death, if infected) were associated with higher levels of moral distress and two organizational factors (adequacy of access to PPE and perceived organizational support) were associated with lower levels. Fifth, moderate and high levels of moral distress intensity were associated with substantially to markedly increased odds of adverse outcomes: generalized anxiety, depression, PTSD, burnout and intention to leave direct patient care in the next five years.

Results from this study are consistent with most studies conducted during the pandemic, which have generally shown low levels of moral distress intensity, on average, and low frequency of episodes of compromised care among physicians and other health disciplines [11, 13–21]. However, we also found that about 1 in 10 physicians experienced compromised care due to resource limitations frequently and had high levels of moral distress; those with high levels of moral distress had markedly elevated adverse mental health outcomes, burnout and intention to leave patient care.

Our study, like prior studies of moral distress during the COVID-19 pandemic, found strong links between degree of exposure to COVID-19 (and its consequences) and moral distress [13, 14, 16, 19, 25]. Health care workers who have high levels of exposure or risk of death from COVID-19 believe that the pandemic makes it difficult for them to provide optimal patient care because of fear of being infected or dying from COVID-19 [44].

The findings have important implications. Attention should be focused on identifying internists with moderate and high levels of moral distress rather than on all physicians who report any moral distress given the generally low levels of moral distress reported by internists in this study, the null association between low levels of moral distress and four of the five outcomes and the strong associations between moderate/high levels and all of the outcomes. Moral distress of any severity is concerning but low levels are associated only weakly with anxiety but no other outcomes while high levels are associated with dramatically increased odds of all adverse outcomes. Two factors—self-reported risk of death, if infected with COVID and number of patients seen face-to-face with COVID-19—are associated with greater moral distress and might be useful for identifying physicians at risk of high levels of moral distress.

Resource limitations that compromise patient care are bad for patients but our study suggests that they are bad for physicians as well. Health care workers in other studies have reported that compromised patient care due to resource limitations is one of the most morally distressing situations that they had encountered during the COVID-19 pandemic [11, 13]. Although few health organizations in the U.S. are currently reporting the severe shortages in personal protective equipment that marked the early stages of the pandemic [45], shortages in beds, staffing, and equipment related to the surges driven by the SARS-CoV-2 delta variant and omicron variant are now common and may be associated with compromised care [46, 47]. Our finding that compromised care during the worst period of the pandemic was associated with current moral distress suggests that the impact on physicians may be long lasting.

Many interventions have been proposed to address moral distress related to the COVID-19 pandemic though none of them have been evaluated and demonstrated to prevent or treat moral distress, when present [12, 13, 48–51]. Although our study was not an intervention study, we found that perceived organizational support was associated with substantially lower levels of moral distress. This finding is consistent with those from a systematic review of multiple studies conducted prior to the pandemic demonstrating inverse associations between supportive ethical health care organizational climate and moral distress [3]. Meta-analyses in organizations of all types, including health organizations, have identified specific actions that organizations can take to foster perceived organizational support among their employees [52, 53]. Implementation of those actions might reduce moral distress by increasing perceived organizational support.

This study had a number of limitations. The study design was cross-sectional. It is unclear whether the findings, obtained from a survey conducted in September and October of 2020 would be the same if the survey had been conducted at other times during the pandemic. The cross-sectional design also makes directional causal inference impossible; we have assumed that mental health outcomes, burnout and intention to leave clinical practice are consequences of moral distress but it is possible that they are causes of moral distress. A recently published longitudinal study demonstrated that current moral distress predicted future burnout and future mental strain, a composite of anxiety and depression [23]. This provides some support for the direction of causality we assumed in this study, though additional longitudinal research is needed. Additionally, the response rate of 37.8% is lower than ideal for survey research but is substantially higher than other large studies of moral distress during the pandemic [13, 14]. Unlike studies that used convenience sampling [9, 10, 16–23, 25], we were able to assess and adjust, through post-stratification and raking, for differences in demographic characteristics between the sample and the population being sampled. Finally, we measured moral distress with a single-item scale that has good, but imperfect association with multi-item scales that have been used to measure moral distress [28]. However, error in the measurement of moral distress is likely to be non-differential with respect to both predictors and putative outcomes of moral distress, leading to an attenuation of the reported strength of those associations [54].

The study also had notable strengths. It is the first national study of moral distress related to the COVID-19 pandemic in the United States. Our findings go beyond other studies of moral distress during the pandemic by demonstrating that moderate and high levels of moral distress, but not low ones, are likely to be clinically significant. We also identified a factor, perceived organizational support, that can be influenced by health care organizational actions and that may lower moral distress. Future research should assess longitudinal trajectories of moral distress and its impact on long-term adverse outcomes and evaluate, through intervention research, the impact of actions designed to increase perceived organizational support on moral distress.

## Supporting information

S1 File Data set, variable definitions and response categories. (XLSX)

S2 File MPLUS code for multiple linear regression of moral distress on all predictors, adjusted for demographic covariates. (DOCX)

S3 File MPLUS code for multiple logistic regression associations between moral distress (coded as three indicator variables) and five outcomes. (DOCX)

S4 File Multiple linear regression for associations between individual and organizational predictors of moral distress, adjusted for potential confounding factors. (DOCX)

S5 File Multiple logistic regression results for screening positive for anxiety, depression, PTSD, high burnout and intention to leave patient care. (DOCX)

S6 File Moral distress thermometer. (DOCX)
