# Supplementary material for: A national study of moral distress among U.S. internal medicine physicians during the COVID-19 pandemic
Source: PLoS One. 2022 May 16;17(5):e0268375. doi: 10.1371/journal.pone.0268375 (PMC9109912; doi:10.1371/journal.pone.0268375)
Supplement: S2 File — (DOCX) [file pone.0268375.s002.docx]

**S2 File. MPLUS Code for multiple linear regression of moral distress on all predictors, adjusted for demographic covariates** (Last two columns of Table 1).

TITLE: ACP COVID REGRESSIONS ALL

DATA: File is "{your path} \moral_distress.txt”

VARIABLE: NAMES ARE

ftf

ftf_four

hrs_cat

cov_risk

cov_die

ipt_otpt

ppe

com_list

moral32

moral32_high

moral33

moral33_high

ORG35

age

liv_hom

spec_rsk

race_rev

wt7

perc_sup

warn

anxiety

depression

ptsd

burn_h

leave_h

reg1

reg2

reg4

race2

race3

race4

race5

;

MISSING = ALL(999);

Usevariables are

ftf_four

cov_risk

cov_die

ipt_otpt

ppe

com_list

ORG35

age

liv_hm

spec_rsk

hrs_cat

wt7

gend_rev

perc_sup

warn_rev

reg1

reg2

reg4

race2

race3

race4

race5

;

weight is wt7;

ANALYSIS:

algorithm = integration;

integration = montecarlo;

MODEL:

Moral32 on

ftf_four

cov_risk

cov_die

ipt_otpt

ppe

com_list

ORG35

age

liv_hm

spec_rsk

hrs_cat

wt7

gend_rev

perc_sup

warn_rev

reg1

reg2

reg4

race2

race3

race4

race5

;

ftf_four

cov_risk

cov_die

ipt_otpt

ppe

com_list

ORG35

age

liv_hm

spec_rsk

hrs_cat

wt7

gend_rev

perc_sup

warn_rev

reg1

reg2

reg4

race2

race3

race4

race5

;

Output:

Standardized (STDYX);
