# Supplementary material for: A national study of moral distress among U.S. internal medicine physicians during the COVID-19 pandemic
Source: PLoS One. 2022 May 16;17(5):e0268375. doi: 10.1371/journal.pone.0268375 (PMC9109912; doi:10.1371/journal.pone.0268375)
Supplement: S3 File — (DOCX) [file pone.0268375.s003.docx]

**S3 File.** **MPLUS Code for multiple logistic regression associations between moral distress intensity (coded as three indicator variables) and anxiety.**

For the other four outcomes (depression, PTSD, burnout and intention to leave patient care), substitute those outcomes for anxiety.

Reproduces data in Table 2

DATA: File is "{your path} \moral_distress.txt";

VARIABLE: NAMES ARE

ftf

cov_risk

cov_die

ipt_otpt

ppe

com_list

moral32

moral33

ORG35

age

liv_hm

spec_rsk

hrs_tot

race_rev

wt7

gend_rev

perc_sup

warn

depression

anxiety

ptsd

burn_H

mor33_h

reg1

reg2

reg4

leave_h

race2

race3

race4

race5

newid

ftf_quart

hrs_cat

mor32_h

moral32_ord

mor_dum1

mor_dum2

mor_dum3

;

MISSING = ALL(999);

Usevariables are

anxiety mor_dum1 mor_dum2 mor_dum3

ftf

age liv_hm gend_rev

race2 race3 race4 race5

cov_risk cov_die ipt_otpt

reg1 reg2 reg4

;

weight is wt7;

categorical is anxiety;

DEFINE:

Cut ftf (0, 9, 19);

ANALYSIS:

algorithm = integration;

integration = montecarlo;

MODEL: anxiety on mor_dum1 mor_dum2 mor_dum3

ftf

age liv_hm gend_rev

race2 race3 race4 race5

cov_risk cov_die ipt_otpt

reg1 reg2 reg4

;

mor_dum1 mor_dum2 mor_dum3

ftf

age liv_hm gend_rev

race2 race3 race4 race5

cov_risk cov_die ipt_otpt

reg1 reg2 reg4

;
