# Supplementary material for: A national study of moral distress among U.S. internal medicine physicians during the COVID-19 pandemic
Source: PLoS One. 2022 May 16;17(5):e0268375. doi: 10.1371/journal.pone.0268375 (PMC9109912; doi:10.1371/journal.pone.0268375)
Supplement: S4 File — (DOCX) [file pone.0268375.s004.docx]

**S4 File. Multiple Linear Regression for Associations between individual and organizational predictors of moral distress, adjusted for potential confounding factors. Analyses conducted in Mplus version 8.5.**

R-SQUARE

Variable **Estimate S.E. Est./S.E. P-Value**

MORAL32 0.262 0.033 7.827 0.000

MODEL FIT INFORMATION

Number of Free Parameters 275

Loglikelihood

H0 Value -16298.589

H0 Scaling Correction Factor 1.2506

for MLR

Information Criteria

Akaike (AIC) 33147.178

Bayesian (BIC) 34438.862

Sample-Size Adjusted BIC 33565.576

(n* = (n + 2) / 24)

MODEL UNSTANDARDIZED RESULTS

MORAL32 ON **Est. SE Est. / SE P value**

IPT_OTPT -0.160 0.152 -1.050 0.294

SPEC_RSK -0.052 0.256 -0.203 0.839

FTF 0.385 0.095 4.058 0.000

COV_RISK -0.004 0.093 -0.042 0.967

COV_DIE 0.705 0.114 6.188 0.000

PPE -0.190 0.084 -2.269 0.023

COM_LIST -0.078 0.127 -0.616 0.538

PERC_SUP -0.138 0.032 -4.302 0.000

ORG35 -0.007 0.186 -0.039 0.969

WARN 0.048 0.283 0.170 0.865

AGE -0.211 0.075 -2.822 0.005

LIV_HM 0.079 0.060 1.325 0.185

HRS_TOT 0.125 0.065 1.927 0.054

GEND_REV 0.122 0.169 0.721 0.471

REG1 0.001 0.226 0.003 0.997

REG2 -0.169 0.208 -0.815 0.415

REG4 0.332 0.207 1.609 0.108

RACE2 0.309 0.187 1.654 0.098

RACE3 -0.411 0.407 -1.009 0.313

RACE4 0.355 0.340 1.045 0.296

RACE5 0.010 0.281 0.037 0.971

MODEL *STANDARDIZED* RESULTS (Reported in Table 1 for predictors, underlined below)

MORAL32 ON **Est. SE Est. / SE P value**

IPT_OTPT -0.054 0.051 -1.053 0.292

SPEC_RSK -0.010 0.050 -0.203 0.839

FTF 0.154 0.037 4.141 0.000

COV_RISK -0.002 0.039 -0.042 0.967

COV_DIE 0.265 0.042 6.275 0.000

PPE -0.092 0.040 -2.280 0.023

COM_LIST -0.032 0.052 -0.616 0.538

PERC_SUP -0.221 0.051 -4.288 0.000

ORG35 -0.001 0.037 -0.039 0.969

WARN 0.006 0.037 0.170 0.865

AGE -0.107 0.037 -2.865 0.004

LIV_HM 0.049 0.037 1.328 0.184

HRS_TOT 0.077 0.040 1.938 0.053

GEND_REV 0.026 0.036 0.721 0.471

REG1 0.000 0.040 0.003 0.997

REG2 -0.030 0.037 -0.815 0.415

REG4 0.061 0.038 1.612 0.107

RACE2 0.061 0.037 1.656 0.098

RACE3 -0.040 0.040 -0.998 0.318

RACE4 0.037 0.035 1.056 0.291

RACE5 0.001 0.031 0.037 0.971
