# Supplementary material for: A national study of moral distress among U.S. internal medicine physicians during the COVID-19 pandemic
Source: PLoS One. 2022 May 16;17(5):e0268375. doi: 10.1371/journal.pone.0268375 (PMC9109912; doi:10.1371/journal.pone.0268375)
Supplement: S5 File — (DOCX) [file pone.0268375.s005.docx]

**S5 File. Multiple Logistic Regression Results for Screening Positive for Anxiety, Depression, PTSD, High Burnout and Intention to Leave Patient Care**

**All analyses conducted in Mplus version 8.5**

**1. Regression Results for Multiple Logistic Regression of Screening Positive for Anxiety on Moral Distress Indicator Variables, Controlling for Potential Confounding Factors**

MODEL FIT INFORMATION

Number of Free Parameters 188

Loglikelihood H0 Value -9701.071

H0 Scaling Correction Factor 1.2322

for MLR

Information Criteria

Akaike (AIC) 19778.143

Bayesian (BIC) 20661.185

Sample-Size Adjusted BIC 20064.175

(n* = (n + 2) / 24)

MODEL RESULTS

ANXIETY ON **Est. SE Est/SE P value**

MOR_DUM1 0.865 0.427 2.028 0.043

MOR_DUM2 1.583 0.445 3.558 0.000

MOR_DUM3 2.343 0.441 5.314 0.000

FTF 0.105 0.141 0.746 0.456

AGE -0.102 0.114 -0.897 0.370

LIV_HM 0.054 0.081 0.665 0.506

GEND_REV 0.817 0.244 3.348 0.001

RACE2 -0.269 0.281 -0.957 0.339

RACE3 0.584 0.635 0.920 0.358

RACE4 0.191 0.456 0.419 0.675

RACE5 -0.325 0.449 -0.725 0.469

COV_RISK 0.064 0.141 0.458 0.647

COV_DIE 0.291 0.164 1.774 0.076

IPT_OTPT 0.146 0.160 0.911 0.363

REG1 -0.005 0.330 -0.016 0.987

REG2 -0.788 0.369 -2.132 0.033

REG4 -0.226 0.307 -0.734 0.463

**2. Regression Results for Multiple Logistic Regression of Screening Positive for Depression on Moral Distress Indicator Variables, Controlling for Potential Confounding Factors**

MODEL FIT INFORMATION

Number of Free Parameters 188

Loglikelihood

H0 Value -9665.040

H0 Scaling Correction Factor 1.2246

for MLR

Information Criteria

Akaike (AIC) 19706.080

Bayesian (BIC) 20589.122

Sample-Size Adjusted BIC 19992.112

(n* = (n + 2) / 24)

MODEL RESULTS

DEPRESSION ON **Est. SE Est/SE P val**

MOR_DUM1 0.104 0.394 0.263 0.792

MOR_DUM2 0.859 0.414 2.073 0.038

MOR_DUM3 1.463 0.416 3.513 0.000

FTF -0.103 0.163 -0.631 0.528

AGE -0.122 0.119 -1.026 0.305

LIV_HM -0.070 0.088 -0.791 0.429

GEND_REV -0.323 0.279 -1.160 0.246

RACE2 -0.095 0.295 -0.322 0.747

RACE3 0.702 0.631 1.113 0.266

RACE4 -0.608 0.552 -1.102 0.271

RACE5 -0.216 0.430 -0.504 0.615

COV_RISK 0.248 0.155 1.604 0.109

COV_DIE 0.476 0.174 2.735 0.006

IPT_OTPT 0.037 0.183 0.201 0.841

REG1 0.076 0.367 0.208 0.835

REG2 -0.701 0.405 -1.730 0.084

REG4 0.222 0.322 0.691 0.489

**3. Regression Results for Multiple Logistic Regression of Screening Positive for PTSD on Moral Distress Indicator Variables, Controlling for Potential Confounding Factors**

MODEL FIT INFORMATION

Number of Free Parameters 188

Loglikelihood

H0 Value -9659.262

H0 Scaling Correction Factor 1.2222

for MLR

Information Criteria

Akaike (AIC) 19694.524

Bayesian (BIC) 20577.566

Sample-Size Adjusted BIC 19980.556

(n* = (n + 2) / 24)

MODEL RESULTS

Two-Tailed

Estimate S.E. Est./S.E. P-Value

PTSD ON **Est. SE Est / SE P value**

MOR_DUM1 0.583 0.519 1.122 0.262

MOR_DUM2 1.714 0.494 3.471 0.001

MOR_DUM3 2.441 0.515 4.741 0.000

FTF 0.004 0.144 0.031 0.975

AGE -0.131 0.118 -1.111 0.267

LIV_HM 0.062 0.085 0.728 0.466

GEND_REV 0.006 0.261 0.021 0.983

RACE2 0.265 0.283 0.935 0.350

RACE3 -0.773 0.857 -0.902 0.367

RACE4 -0.250 0.629 -0.397 0.691

RACE5 0.030 0.457 0.065 0.948

COV_RISK 0.077 0.153 0.505 0.613

COV_DIE 0.562 0.172 3.277 0.001

IPT_OTPT 0.092 0.175 0.525 0.599

REG1 -0.010 0.357 -0.029 0.977

REG2 -1.077 0.408 -2.640 0.008

REG4 -0.046 0.291 -0.157 0.875

**4. Regression Results for Multiple Logistic Regression of Screening Positive for High Burnout on Moral Distress Indicator Variables, Controlling for Potential Confounding Factors**

MODEL FIT INFORMATION

Number of Free Parameters 188

Loglikelihood

H0 Value -9831.888

H0 Scaling Correction Factor 1.2256

for MLR

Information Criteria

Akaike (AIC) 20039.777

Bayesian (BIC) 20922.819

Sample-Size Adjusted BIC 20325.809

(n* = (n + 2) / 24)

MODEL RESULTS

BURN_H ON **Est. SE Est / SE P value**

MOR_DUM1 0.143 0.252 0.569 0.569

MOR_DUM2 0.718 0.275 2.611 0.009

MOR_DUM3 1.992 0.314 6.343 0.000

FTF -0.138 0.113 -1.224 0.221

AGE -0.027 0.090 -0.304 0.761

LIV_HM 0.045 0.064 0.692 0.489

GEND_REV 0.555 0.194 2.858 0.004

RACE2 -0.502 0.227 -2.208 0.027

RACE3 -0.490 0.578 -0.848 0.396

RACE4 -0.363 0.449 -0.808 0.419

RACE5 -0.101 0.326 -0.310 0.756

COV_RISK 0.183 0.111 1.638 0.101

COV_DIE 0.242 0.127 1.899 0.058

IPT_OTPT -0.291 0.136 -2.149 0.032

REG1 0.299 0.253 1.183 0.237

REG2 0.021 0.266 0.080 0.936

REG4 0.048 0.246 0.196 0.845

**5. Regression Results for Multiple Logistic Regression of Screening Positive for Intention to Leave Patient Care on Moral Distress Indicator Variables, Controlling for Potential Confounding Factors**

MODEL FIT INFORMATION

Number of Free Parameters 188

Loglikelihood

H0 Value -9751.335

H0 Scaling Correction Factor 1.2206

for MLR

Information Criteria

Akaike (AIC) 19878.670

Bayesian (BIC) 20761.712

Sample-Size Adjusted BIC 20164.702

(n* = (n + 2) / 24)

MODEL RESULTS

LEAVE_H ON **Est. SE Est / SE P value**

MOR_DUM1 0.131 0.258 0.507 0.612

MOR_DUM2 0.109 0.308 0.353 0.724

MOR_DUM3 1.093 0.335 3.264 0.001

FTF 0.147 0.124 1.193 0.233

AGE 0.390 0.102 3.804 0.000

LIV_HM -0.286 0.075 -3.803 0.000

GEND_REV -0.021 0.209 -0.099 0.921

RACE2 -0.128 0.249 -0.513 0.608

RACE3 -1.162 0.666 -1.745 0.081

RACE4 -0.640 0.640 -1.000 0.317

RACE5 -0.084 0.350 -0.241 0.810

COV_RISK 0.024 0.133 0.180 0.857

COV_DIE 0.415 0.143 2.911 0.004

IPT_OTPT -0.140 0.153 -0.917 0.359

REG1 0.273 0.282 0.970 0.332

REG2 0.277 0.295 0.940 0.347

REG4 0.521 0.280 1.864 0.062
