# Supplementary material for: A national study of moral distress among U.S. internal medicine physicians during the COVID-19 pandemic
Source: PLoS One. 2022 May 16;17(5):e0268375. doi: 10.1371/journal.pone.0268375 (PMC9109912; doi:10.1371/journal.pone.0268375)
Supplement: S6 File — (DOCX) [file pone.0268375.s006.docx]

**S6 File. Moral Distress Thermometer**

Moral distress is a form of distress that occurs when you believe you know the ethically correct thing to do, but something or someone restricts your ability to pursue the right course of action. Please indicate below the number that best describes how much moral distress you have been experiencing related to work in the past two weeks including today.

______________

🞊 10 Worst possible

🞊 9

🞊 8 Intense

🞊 7

🞊 6 Distressing

🞊 5

🞊 4 Uncomfortable

🞊 3

🞊 2 Mild

🞊 1

🞊 0 None
